# Supplementary material for: Selenium Nanoparticles Suppressed Oxidative Stress and Promoted Tenocyte Marker Expression in Tendon-Derived Stem/Progenitor Cells
Source: Antioxidants (Basel). 2024 Dec 15;13(12):1536. doi: 10.3390/antiox13121536 (PMC11727164; doi:10.3390/antiox13121536)
Supplement: Supplementary file 1 [file antioxidants-13-01536-s001.zip › antioxidants-3374530-supplementary.pdf]

Table S1. Primer sequence of qRT-PCR

Gene	Primer sequence	Accession number
Nox1	(F) 5′ CCCCTGAGTCTTGGAAGTGG 3′<br>(R) 5′ GGGTGCATGACAACCTTGG 3′	NM_053683.2
Gpx1	(F) 5′ CAGTTCGGACATCAGGAGAATGG 3′<br>(R) 5′ CGCAGGAAGGTAAAGAGCG 3′	NM_030826.4
Gpx3	(F) 5′ GTCTCAAGTACGTTCGACCGG 3′<br>(R) 5′ GAGGGCAGGAGTTCTTCAGG 3′	NM_022525.4
Gpx4	(F) 5′ GGGCTTGTGTGCATCCC 3′<br>(R) 5′ ACGCAACCCCTGTACTTATCC 3′	NM_017165.4
Hmox1	(F) 5′ GCTCAACATTGAGCTGTTTGAGG 3′<br>(R) 5′ GTATCTTGAACCAGGCTAGCAGG 3′	NM_012580.2
Txnrd2	(F) 5′ TACGGCTGGGAGGTGGC 3′<br>(R) 5′ CCTGAAGTTGGACACGATGACC	NM_022584.3
Selenom	(F) 5′ GGGTGGAGACCTGTGGAGG 3′<br>(R) 5′ GAGTGGGATTCGCTCTAGTTCC 3′	NM_001115013.2
Sod1	(F) 5′ GCGGTGAACCAGTTGTGGTG 3′<br>(R) 5′AGCCACATTGCCCAGGTCTCS 3′	NM_012520.2
Cat	(F) 5′ ACTGGGACCTCGTGGGAAAC 3′<br>(R) 5′ TCTGGAATCCCTCGGTCGCT 3′	NM_012520.2
Il6	(F) 5′ TCCTACCCCAACTTCCAATGCTC 3′<br>(R) 5′ TTGGATGGTCTTGGTCCTTAGCC 3′	NM_012589.2
Cox2/Ptgs2	(F) 5′ TGTATGCTACCATCTGGCTTCGG 3′<br>(R) 5′ GTTTGGAACAGTCGCTCGTCATC 3′	S67722.1
Il1b	(F) 5′ CACCTCTCAAGCAGAGCACAG 3′<br>(R) 5′ GGGTTCCATGGTGAAGTCAAC 3′	NM_031512.2
Bax	(F) 5' GCTACAGGGTTTCATCCAGGATC 3′<br>(R) 5' CAGCAATCATCCTCTGCAGC 3′	NM_017059.2
Bad	(F) 5' GAATGAGCGATGAATTTGAGGGT 3′<br>(R) 5' CCTTTCCCCAAATTTCGATCCC 3′	NM_022698.2
Bid	(F) 5′ TGGACTCTGAGGTCAGCAATG 3′<br>(R) 5′ TTCGGAGAAAGCCGAACACC 3′	NM_022684.2
Casp3	(F) 5′ GTGGAACTGACGATGATATGGC 3′<br>(R) 5′ CGCAAAGTGACTGGATGAACC 3′	NM_012922.2
Bcl2l1	(F) 5′ GCATTGTGGCCTTCTTCTCC 3′<br>(R) 5′ CAACTTGCAATCCGACTCACC 3′	NM_001033672.1
Bcl2	(F) 5′ GTGGATGACTGAGTACCTGAACC 3′<br>(R) 5′ CAGCCAGGAGAAATCAAACAGAG 3′	NM_016993.2
Col1a1	(F) 5′ CATCGGTGGTACTAAC 3′<br>(R) 5′ CTGGATCATATTGCACA 3′	NM_053356.1
Col3a1	(F) 5′ TGCAATGTGGGACCTGGTTT 3′<br>(R) 5′ GGGCAGTCTAGTGGCTCATC 3′	NM_032085.1
Eln	(F) 5′ GCTTAGGAGTCTCAACAGGTGC 3′<br>(R) 5′ CGGAACCTTGGCCTTGACTC 3′	NM_012722.1
Tnc	(F) 5′ AAAGCAGCCACCCGCTATTAC 3′<br>(R) 5′ GGATCTCCTCTGTCAAGACCTCAA 3′	NM_053861.2

Dcn	(F) 5′ GTTCTGATCTGGGTCTGGACAAAG 3′<br>(R) 5′ CTTAAAGGCCCCCTCTTTGATC 3′	NM_024129.1
Nrf2	(F) 5′ CATTTGTAGATGACCATGAGTCGC 3′<br>(R) 5′ ATCAGGGGTGGTGAAGACTG 3′	NM_031789.2
Sirt1	(F) 5′ CCAGATTTCAAGGCTGTTGGTTCC 3′<br>(R) 5′ CCACAGGAACTAGAGGATAAGGCGT 3′	XM_041656502.1
FoxO1	(F) 5′ TCAGGCTAGGAGTTAGTGAGCA 3′<br>(R) 5′ GGGGTGAAGGGCATCTTT 3′	XM_039103268.2
Actb	(F) 5′ CAGGGTGTGATGGTGGGTATGG 3′<br>(R) 5′ AGTTGGTGACAATGCCGTGTTC 3′	NM_031144.3
